# Multistage pig identification using a sequential ear tag detection pipeline

**DOI:** 10.1038/s41598-025-05283-8

**Published:** 2025-06-20

**Authors:** Martin Wutke, Damiano Debiasi, Shobhana Tomar, Jeanette Probst, Nicole Kemper, Kai Gevers, Marc-Alexander Lieboldt, Imke Traulsen

**Affiliations:** 1 Institute of Animal Breeding and Husbandry, Faculty of Agricultural and Nutritional Sciences, Christian-Albrechts-University Kiel, Kiel, 24118 Germany; 2 Institute for Animal Hygiene, Animal Welfare and Farm Animal Behavior (ITTN), University of Veterinary Medicine Hannover, Foundation, Hannover, Germany; 3 Chamber of Agriculture Lower Saxony, Division Agriculture, Oldenburg, Oldenburg, Germany

**Keywords:** Animal identification, Computer vision, YOLOv10, Animal behaviour, Machine learning

## Abstract

Reliable animal identification in livestock husbandry is essential for various applications, including behavioral monitoring, welfare assessment, and the analysis of social structures. Although recent advancements in deep learning models have improved animal identification using biometric markers, their applicability remains limited for species without distinctive traits like pigs. Consequently, synthetic features such as ear tags have become widely adopted. However, challenges such as poor lighting conditions and the complexity of ear tag coding continue to restrict the effectiveness of Computer Vision and Deep Learning techniques. In this study, we introduce a robust, lighting-invariant method for individual pig identification that leverages commercially available ear tags within a sequential detection pipeline. Our approach employs four object detection models in succession to detect pigs, localize ear tags, perform rotation correction via pin detection, and recognize digits, ultimately generating a reliable ID proposal. In a first evaluation stage, we assessed the performance of each model independently, achieving a mAP0.95 value of 0.970, 0.979, 0.974 and 0.979 for the pig detection, ear tag detection, pin detection and ID classification model, respectively. In addition, our method was further evaluated in two different camera environments to assess its performance in both familiar and unfamiliar conditions. The results demonstrate that the proposed approach achieves a very high precision of 0.996 in a familiar top-down camera scenario and maintained a strong generalization performance in an unfamiliar, close-up setup with a precision of 0.913 and a recall of 0.903. Furthermore, by publicly proposing three custom datasets for ear tag, pin, and digit detection, we aim to support reproducibility and further research in automated animal identification for precision livestock farming. The findings of this study demonstrate the effectiveness of ID-based animal identification and the proposed method could be integrated within advanced multi-object tracking systems to enable continuous animal observation and for monitoring specific target areas, thereby significantly enhancing overall livestock management systems.

## Introduction

Modern livestock farming is characterized by an increasing animal-to-staff ratio and a growing use of both manual and sensor-based data, which emphasizes the importance of gathering information specific to individual animals. In recent years, many studies have focused on automatic animal identification methods using various approaches for instance the recognition of biometrical features like fur characteristics^1,2^ and facial patterns^3,4^ or the usage of RFID technology^5,6^. However, these identification methods also have certain limitations. Biometric approaches are ineffective for animals that lack distinctive fur markings or body characteristics such as pigs of frequently commercially used genetics. Additionally, proposed methods for facial recognition often require a predefined camera angle for accurate identification, which can be challenging in dynamic farm environments and limits the scope of this approach in terms of generalization. Furthermore, although RFID technology is effective for automated identification in many applications–such as access control and gating systems–its utility is limited by a short functional range, necessitating that animals remain in close proximity to the reader, which diminishes its suitability for continuously monitoring of free-ranging species. In this regard, many studies focus on the application of computer vision (CV) techniques and artificial intelligence (AI) for image-based ear tag identification^7–9^.

In the domain of computer vision and livestock husbandry, the literature highlights two primary research approaches for automatic ear tag identification. The first approach utilizes distinct ear tag coding systems to achieve a bijective form of animal identification. The second approach employs human-readable digit IDs, facilitating both direct animal assignment within the working routine of the farm staff and the potential for automated recognition. Following the first approach, Psota et al. (2020)^7^ introduced an alphanumeric coding system integrated with colored ear tags to enable the identification of individual pigs. While this method attained an average precision exceeding 95%, it was constrained to recognizing only 16 animals due to the combinatorial limitations of the coding system and the fact that the detector had prior knowledge of the total number of animals. Furthermore, Fruhner et al. (2022)^10^ implemented a data matrix coding system to re-identify individual pigs within a conventional housing setup. Although inherently rotationally invariant, this technique encounters difficulties when viewed from a top-down perspective at greater distances, as increased pixelation and blurring negatively impact recognition accuracy. The second approach adopts a more straightforward methodology by employing integer ear tag IDs. In this context, Gao et al. (2024)^11^ propose a vision-based ear tag detection system for dairy cows, utilizing a lightweight Small-YOLOv5 s model to detect ear tags with black digits, alongside a combination of a scene text detection network and a recurrent Network for tag number recognition, aiming to enhance precision livestock management. However, a notable limitation of this approach is the absence of explicit skewness or rotation correction, which can compromise detection accuracy when ear tags are viewed from varying camera angles, potentially leading to an increased rate of false predictions in diverse real-world conditions. Bastiaansen et al. (2022)^8^ focus on real-time cow identification by detecting ear tags in live-stream videos, leveraging yellow color filtering for segmentation and a retrained CNN model for digit recognition. A significant drawback of this method is its dependence on a predefined threshold of yellow pixels to filter detection results, which limits its applicability to environments with controlled lighting and specific camera configurations. Moreover, this color-based filtering approach is ineffective in low-light or nighttime conditions, reducing the system’s reliability for continuous monitoring.

The objective of this study is to overcome the limitations of previous research by introducing a lighting-invariant, image-based animal identification method utilizing commercially available, low-cost ear tags. To achieve this, we propose a processing pipeline in which four distinct object detection models sequentially analyze and filter ear tag information, ultimately producing an ID proposal in the final stage. This work offers two key contributions. First, we present a method that mitigates the shortcomings of existing studies by eliminating dependence on specific color information or intricate ear tag coding systems. Consequently, the proposed approach remains effective for both daytime and nighttime imaging and is adaptable to various scenarios, including both close-range and long-distance identification. Second, due to the scarcity of relevant datasets–particularly for detecting ear tags, pins, and digits–we have developed three distinct datasets and made them publicly available. These datasets can be leveraged to retrain the respective object detection models, ensuring the reproducibility of our approach and fostering further research in this domain.

The remainder of this study is structured as follows. The Materials and methods Section provides a detailed description about the underlying processing pipeline, the data acquisition process and the model training and evaluation rationale. In the Results and Discussion Section, the performance of each detection model is first presented and discussed independently, followed by an holistic assessment of the proposed method for the step wise detection of ear tags and the identification of animal individual IDs using two distinct use case datasets. Finally, this study is concluded by a short summary and an outlook for future research.

## Materials and methods

### Ethics declarations

The study was conducted at the experimental station for pig farming of the Chamber of Agriculture Lower Saxony in Bad Zwischenahn-Wehnen in north-west Germany from March 2024 until September 2024. All experiments were performed and all animals were housed strictly in accordance with European guidelines (EU Council Directive 2008/120/EC), German legislation (German Animal Welfare Act and German Order for the Protection of Production Animals Used for Farming Purposes and Other Animals Kept for the Production of Animal Products) and complied with the ARRIVE guidelines (Animal Research: Reporting of In Vivo Experiments). The trials were reviewed and approved by the Ethics Committee of the Chamber of Agriculture Lower Saxony (approval number: A21-TS21923-LWK-3031-1)

### Data aquisition and processing

**Fig. 1:**
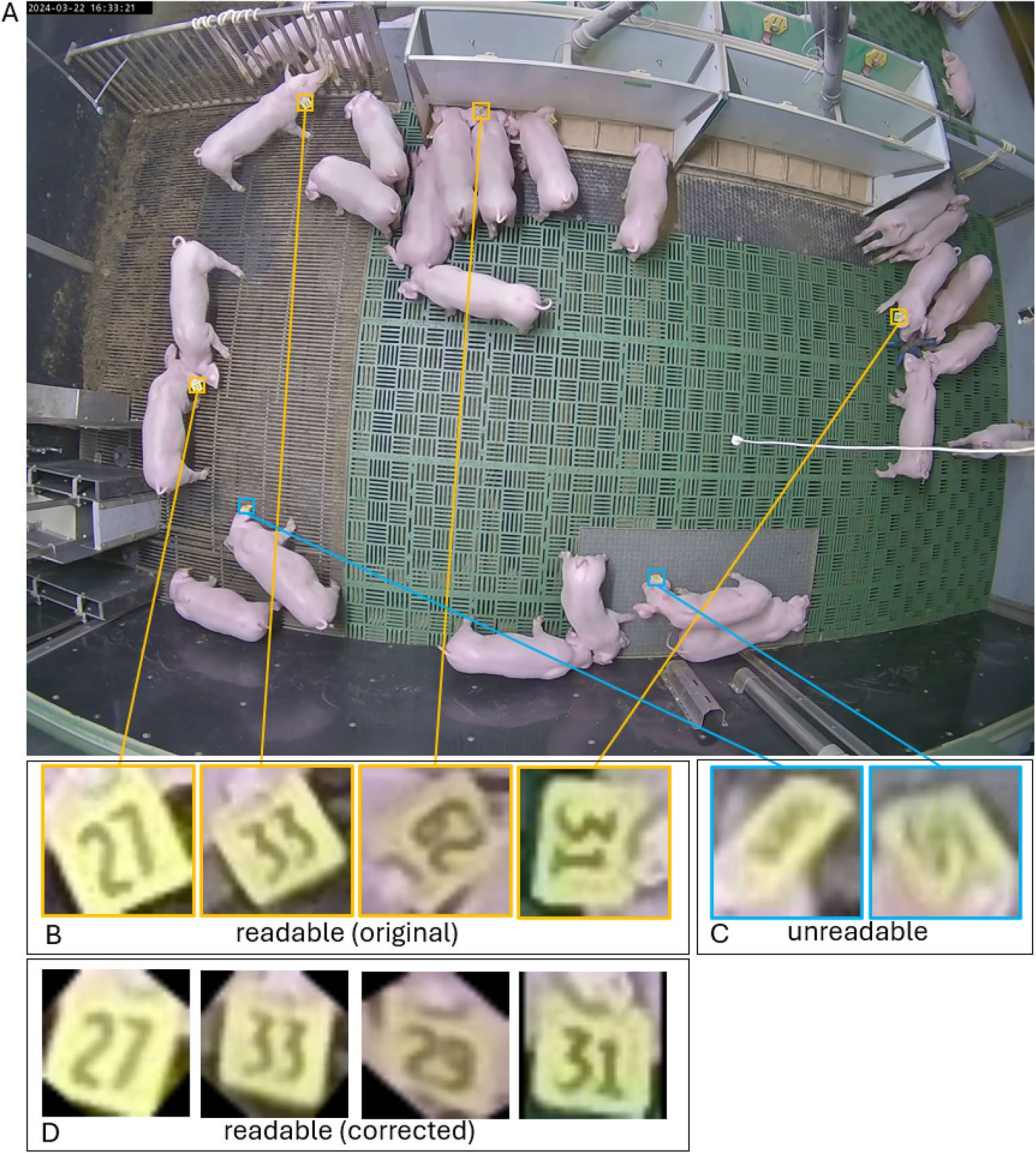
(**A**) Example image of one of the six rearing pens used in this study with four readable (orange) and two unreadabel (blue) ear tags highlighted. (**B**) Four human-readable ear tags before rotation correction. (**C**) Illegible ear tags due to pixelation and occlusion. (**D**) Four human-readable ear tags after processing and rotation correction by the proposed pipeline.

In this study, an extensive video dataset was collected between March 2024 and September 2024 as part of the two collaborative projects DigiSchwein (funding code: 28DE109G18) and Transparency in Pig Production (TiPP) (funding code: 2823ZR016) at the research farm of the Chamber of Agriculture Lower Saxony in Bad Zwischenahn-Wehnen, Germany. Static cameras of the type AXIS M3206-LVE (Axis Communications AB, Lund, Sweden) have been assembled 2.8 m above the ground in each of the six weaning pens used in this study with 24 pigs per pen. The piglets (Pietrain x [Large White x Landrace]) have been born on the farm under conventional housing conditions, and tails were kept undocked. With an average age of 26.48 ± 1.67 days the piglets were moved to the rearing area, where they remained for 39 days. At weaning the piglets received a commercially available yellow ear tag (Primaflex, size 2, Caisley, Bocholt, Germany), which were printed with a two-digit animal ID ranging from 10 to 99. Figure 1 shows an image of a pen area as well as four examples of human readable ear tags and two examples of unreadable ear tags.

During the data collection period, each pen was continuously recorded, with each video file having a runtime of one hour and a frame rate of 20 frames per second (FPS). To enhance processing efficiency and to reduce computation time during model training the original image resolution of $1920^*1080$ pixels was adjusted to $640^*640$ pixels. As the proposed method aims to be operational under practical conditions both during the day and at night, we followed earlier studies and grayscaled each input image to avoid a potential bias between RGB color images and infrared night images^12,13^.

### ID identification framework

**Fig. 2:**
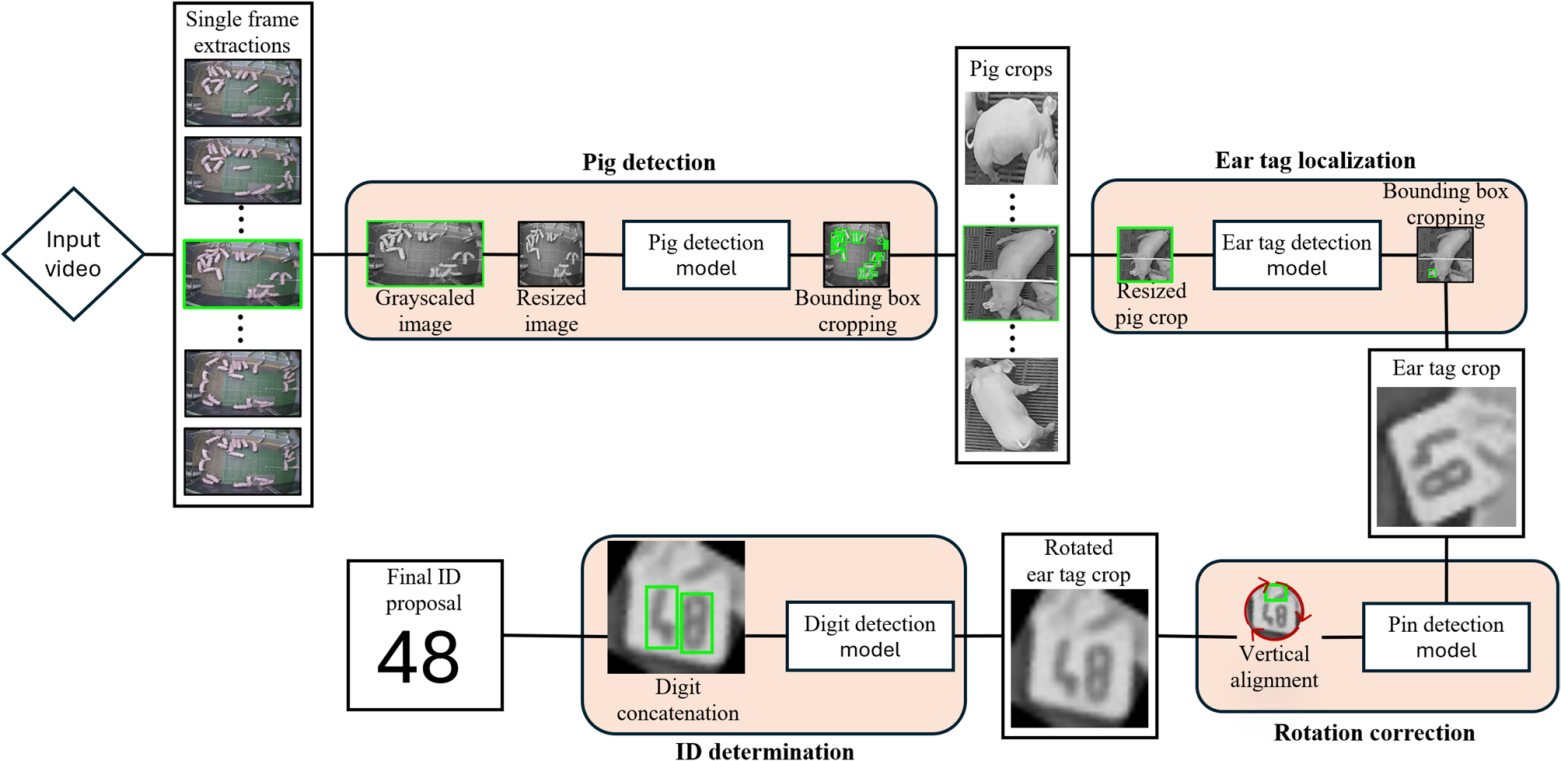
Overview of the proposed analysis pipeline.

In this study, a sequential approach of multiple object detection models is proposed, where the results of a specific model are passed on to the subsequent model for further analysis. In detail, the ear tag identification process consists of four processing stages: (I) individual pig detection, (II) ear tag localization, (III) rotation correction and (IV) ID determination. Since an independent model instance is utilized at each stage, four separate object detection models are used for the overall process, which are hereafter referred to as pig detection model, ear tag detection model, pin detection model and digit detection model. In this regard, the task of object detection refers to the process of locating the position and the class membership of an object of interest within an image or video^14^. Figure 2 illustrates the processing pipeline of the proposed method, where each object detection model targets a specific focus class.

**Fig. 3:**
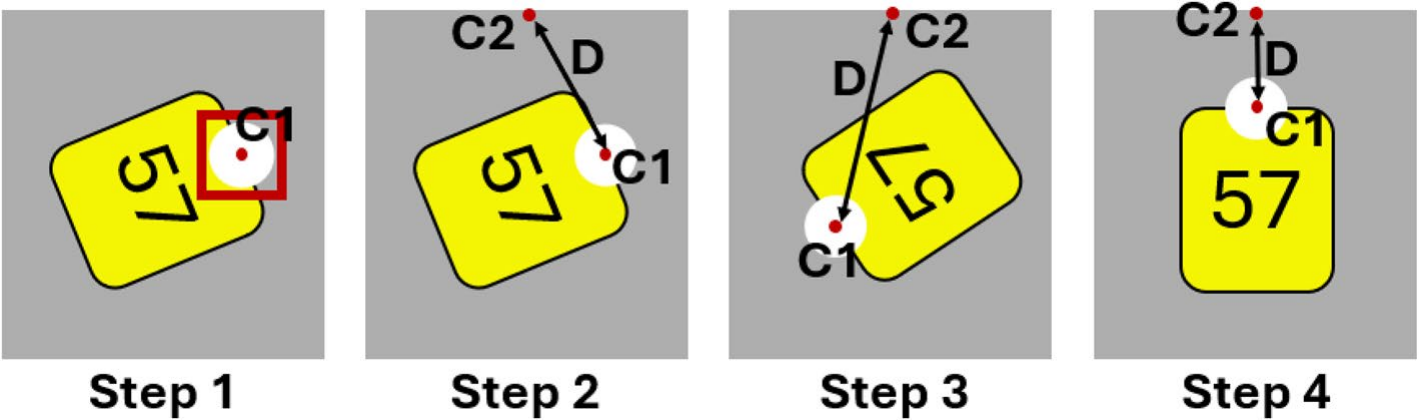
Illustration of a rotation correction for an ear tag with ID “57”. (Step 1) The pin area and the corresponding center point (c1) are detected using the pin detection model. (Step 2) The Euclidean distance (D) between c1 and the center of the upper horizontal image boarder (c2) is calculated. (Step 3 and 4) The ear tag image is rotated until the distance D between c1 and c2 is minimized.

As depicted in Figure 2, the process of ID identification begins with extracting all video frames from the source video and converting each frame into a grayscale format with dimensions of $640^*640$ pixels. This grayscale image is then fed into the pig detection model, which outputs the bounding box coordinates for all visible pigs within the pen. Based on these coordinates, each detected pig is cropped from the original frame to create individual images, each containing a single pig. As the dimensions of these cropped images vary, they are brought back to the squared input dimension. At the second stage, the resized pig images are processed by the ear tag detection model to identify the presence of ear tags. If an ear tag is detected, the model outputs bounding box coordinates, enabling the extraction of a cropped image focused solely on the corresponding ear tag. To ensure the ear tag is properly aligned for the ID identification, the ear tag crop is rotated using the detected ear tag pin, aligning the pin horizontally to the horizontal geometric center of the image by minimizing the Euclidean distance (see Figure 3. In the final stage, the rotation-corrected ear tag image is fed into the digit detection model, which produces bounding box estimates for each digit class, ranging from zero to nine. By arranging the horizontal center coordinates of these estimations in sequential order, the final ID proposal is constructed.

### Datasets

**Fig. 4:**
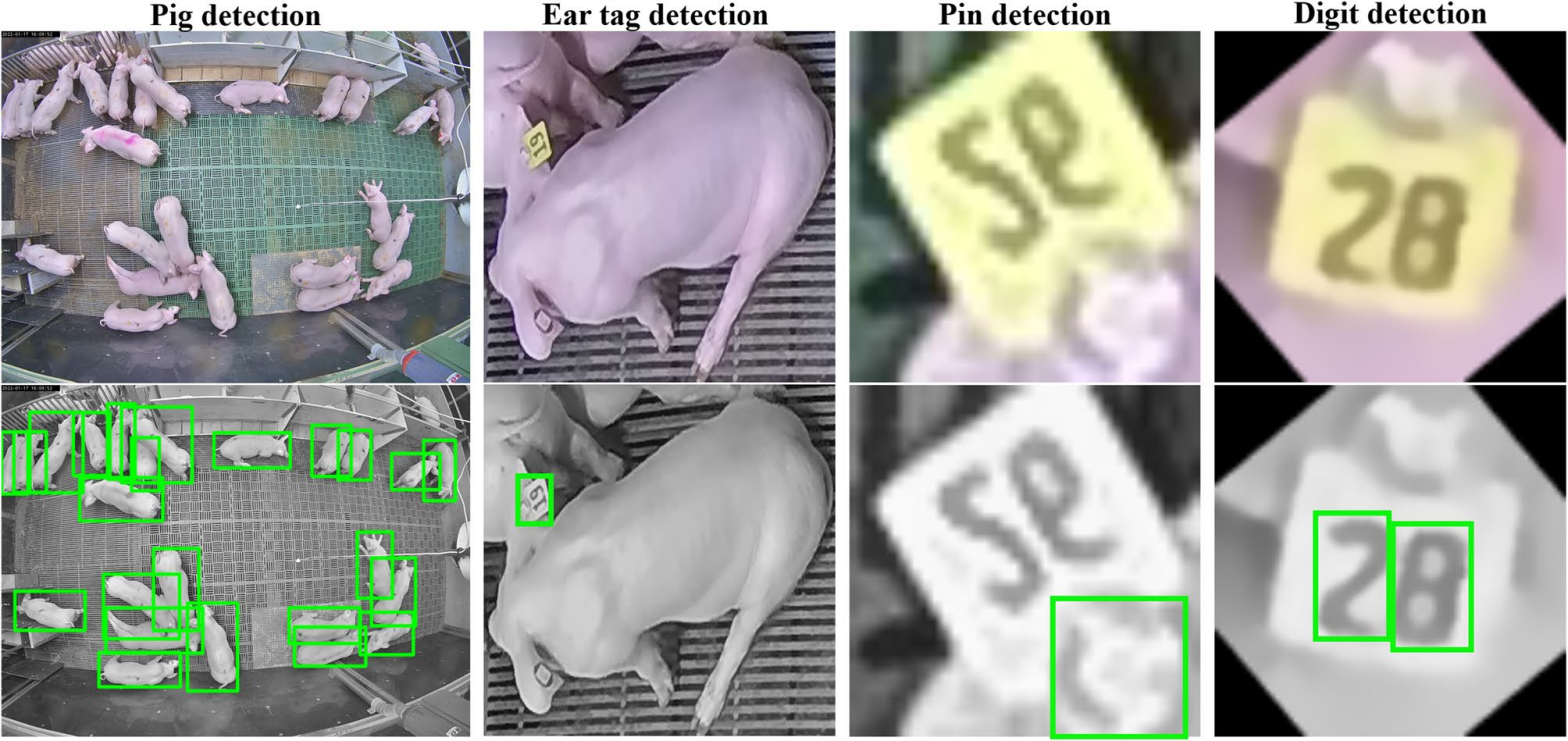
Example images from the individual training datasets used in this study. The top row displays the original RGB color images and the bottom row shows the grayscale images used for model training, with the objects of interest highlighted (green bounding box). While the pig detection dataset published by^15^ is publicly available, the other three datasets were developed in the course of this study.

The following subsection provides an overview of the underlying datasets used in this study. Since the proposed method follows a hierarchical model ensemble strategy using four distinct object detection models, each model was trained and evaluated independently with its own data. Figure 4 presents an example image from each of the four training datasets, organized in the same hierarchical sequence as the model structure employed in this study, starting with the pig detection stage and concluding with the digit detection stage.

The application of object detection techniques in livestock farming has gained considerable attention in recent years. As detecting animal instances is often an initial step in the analysis process, an increasing number of datasets are nowadays available to address various research questions^16^. In the course of this study, the publicly available dataset proposed by Witte et al. (2024) was used for the first processing stage to localize individual animals in the video frame^15^. This pig detection dataset contains a total of 9,281 images from different housing environments, pig breeds, ages and production phases. For each image, the spatial position as well as the associated object class was highlighted using a bounding box annotation. The high heterogeneity of the image material in this diverse dataset therefore allows the adaption of a pig detection model even under changing husbandry environments or breed specific factors. As this dataset was published as part of an earlier study in the DigiSchwein project^15^, the specific pen environment was already included in the data and no additional image annotations had to be generated in the course of this work.

In contrast to the pig detection dataset, which is a more general dataset for detecting whole pig instances across multiple camera environments, the availability of more specific image data is often significantly limited. This scarcity frequently hampers the reproducibility of published studies and necessitates the creation of custom datasets. Similarly, no additional usable image data were available for the present study. Consequently, three extensive datasets were developed as part of the data annotation process to support the subsequent processing steps for ear tag detection, pin detection, and ID determination using a bounding box annotation style. While the ear tag and pin detection datasets are single-class datasets containing 4,103 and 13,776 images, respectively, the ID determination dataset consists of 16,391 images and is structured as a multi-class dataset, with a distinct class assigned to each digit in the range of 0 to 9.

To effectively monitor the learning progress and identify potential overfitting during the training of an object detection model, the underlying dataset is typically partitioned into separate training and validation sets. The training set is used to optimize the model’s parameters, while the validation set allows for an ongoing assessment of its generalization ability throughout the training process. Furthermore, to provide an objective evaluation of the final model performance, an additional independent test set is utilized after training is complete. Following previous studies, we applied a standard dataset splitting approach, using an 8:1:1 ratio to divide all datasets into training, validation, and test subsets^17–19^. Table 1 presents a comprehensive overview of the distribution of images across these subsets, including details on image dimensions and class specifications. An illustration of the training progress using the training and validation sets, as well as the final model evaluation based on the test sets, is provided in the Results Section.

**Table 1:** Data distribution and image information for the individual datasets used in this study, divided into the three subsets used for model training (train), validation (val) and evaluation (test).

Dataset	Nr. of images (train)	Nr. of images (val)	Nr. of images (test)	Image width	Image height	Color mode	Annotated classes
Pig detection	7423	929	929	1920	1280	RGB	pig
Ear tag detection	3281	411	411	640	640	RGB	ear tag
Pin detection	11000	1388	1388	320	320	RGB	pin
Digit detection	13111	1640	1640	120	120	RGB	Integer digits ranging from 0 to 9

To overcome the challenge of limited data accessibility in machine learning and animal science, and to support further research while ensuring the reproducibility of our approach, we have publicly released all three annotated custom datasets developed in this study. By making these datasets available, we aim to facilitate the retraining and optimization of object detection models for ear tag, pin, and digit recognition, thereby contributing to the advancement of automated animal identification methods. The datasets can be accessed at the following link: https://opendata.uni-kiel.de/receive/fdr_mods_00000115?accesskey=p8mePt8lB8RnveYnIjCtR8SO5o8eqot8.

### Model training and evaluation

All models used in this study are trained using the the YOLOv10 object detection framework proposed by Ultralytics^20^ in 2024 with the largest available model version (YOLOv10X). Given the need for fast, accurate, and deployable detection systems in livestock environments, we selected the YOLOv10 architecture as the core of our identification pipeline due to its balance of state-of-the-art performance and real-time efficiency. While its predecessor YOLOv8 remains highly popular due to its strong performance and ease of use, YOLOv10 introduces significant architectural improvements, including the elimination of Non-Maximum Suppression via a dual assignment strategy and an efficiency-accuracy optimized design, resulting in faster and more accurate inference^21^. Compared to transformer-based models like Grounding DINO^22^, which is an effective zero-shot detection approach, YOLOv10 offers lower inference latency and computational overhead, making it more suitable for real-time applications such as livestock monitoring, where deployment on edge devices and practical efficiency can be critical^23^.

The training and evaluation process were conducted on a workstation equipped with an AMD Ryzen Threadripper PRO 5945 WX CPU, 512 GB RAM, and a NVIDIA RTX A6000 GPU. We trained all models with a batch size of 16 images per iteration for 250 epochs, except for the pin detection model, which was extended to 500 epochs based on empirical evidence suggesting improved performance with a longer training duration. We followed previous studies and used the Stochastic Gradient Descent optimizer with a learning rate of 0.01^24–26^. The models have been implemented using the programming language Python (version 3.9), the Deep Learning framework Pytorch (version 2.0.1) and Ubuntu 22.04.3 as the operating system. Each model was trained with various data augmentation techniques including geometric transformations and color space operations like hue, saturation or brightness adjustments, using the default augmentation values. An extensive overview of all augmentations parameters is provided in the Ultralytics documentation^20^.

To assess the computational performance, all video evaluations in this study have been conducted with an FPS rate of one frame in order to take into account the different frame rates of the video data from the respective scenarios. Despite using a sequential model architecture, the proposed method achieves an inference speed of 1.375 frames per second, enabling real-time video analysis without causing any processing delays. Since this study primarily aimed to evaluate the methodological suitability rather than to develop a market-ready, efficiency-optimized application, future enhancements could explore the potential of parallelized approaches in more detail, which may prove particularly beneficial for practical applications on standard consumer devices.

To evaluate the general performance of the proposed method comprehensively, we conducted a two-stage assessment. At the first stage, each model was evaluated independently using its respective test dataset to assess the individual performance of each model. Subsequently, for the second stage we assessed the overall performance of the proposed method for detecting and identifying individual animals within two distinct use case scenarios and specific camera perspectives.

**Fig. 5:**
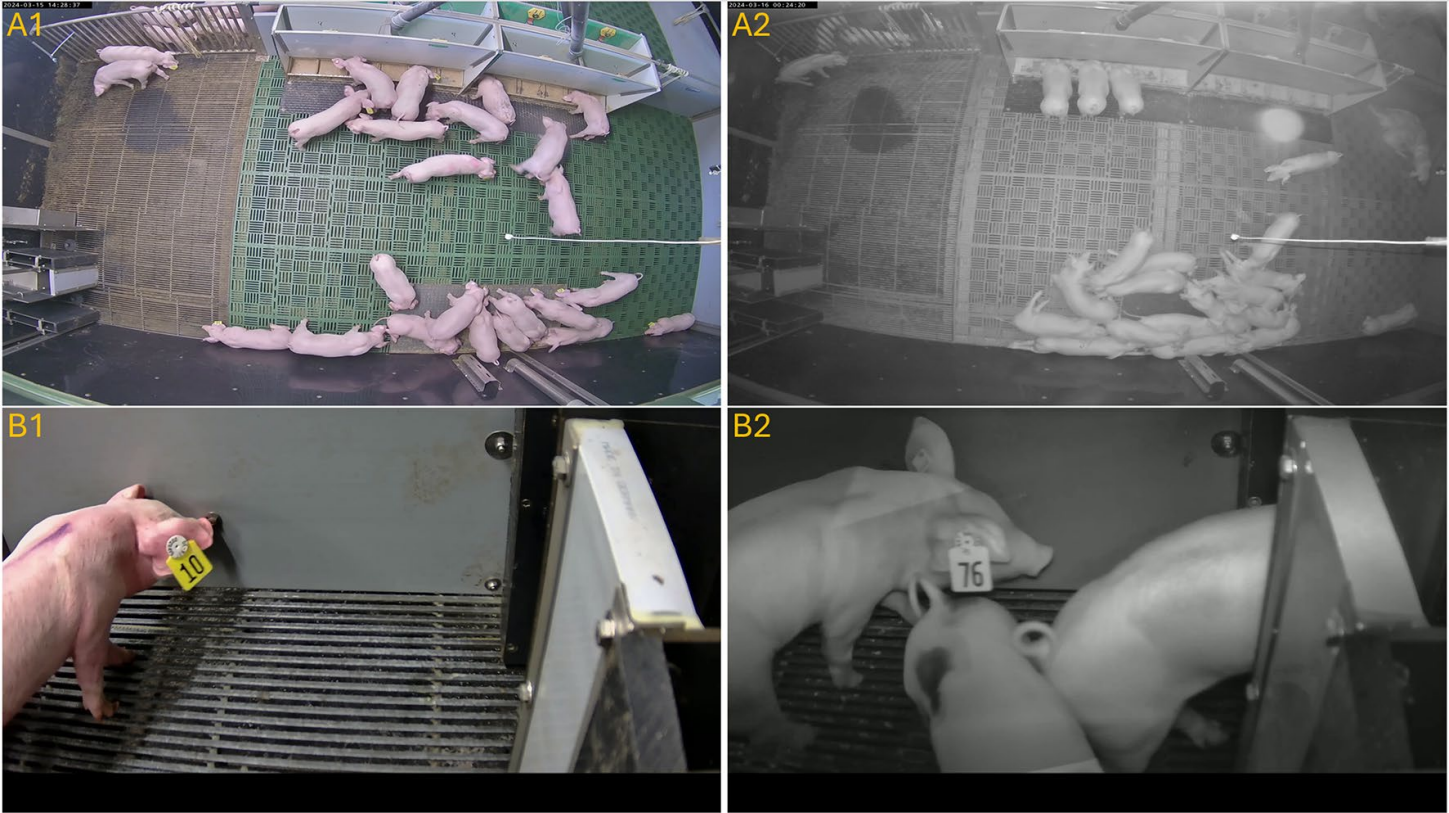
Sample images of a daytime (A1 and B1) and a nighttime (A2 and B2) scene from the first (A1 and A2) and second (B1 and B2) use case scenario, respectively. The images in the second use case show the new camera perspective unknown to the trained models, captured using an autonomous trail camera.

In use case scenario 1, we used new data samples captured from the top-down camera perspective – the same perspective employed during the training of the object detection models (see Figure 1A) – to identify ear tag IDs. In addition, to evaluate the generalizability of our method, the second use case was considered. Here, we collected new video data from the same husbandry environment but a completely new perspective using a common trail camera of the type A323 positioned near a drinking station. This second use case allowed us to assess the method’s ability to perform under conditions not encountered during training, where we kept all model parameter settings the same. Furthermore, the detailed video footage captured by the trail camera enabled us to manually determine the exact number of ear tags that should be detected in each video. This second scenario provided the necessary ground truth data to compute both Recall and Precision values. In addition, since the data and camera perspective from this scenario were not included in any of the training sets, it allowed us to evaluate the level of generalizability of our approach Two example images from both use cases are provided in Figure 5.

For the first evaluation stage, the independent model evaluation, we used the well-established performance metrics Precision, Recall, mean Average Precision (mAP) at an Intersection over Union (IoU) value of 0.5 (mAP0.5) as well as the mAP with an IoU threshold ranging from 0.5 to 0.95, with a step size of 0.05 (mAP:0.95). The metrics are defined as follows:

1 $$\begin{aligned} Recall= & \dfrac{TP}{TP + FN} \end{aligned}$$

2 $$\begin{aligned} Precision= & \dfrac{TP}{TP + FP} \end{aligned}$$

3 $$\begin{aligned} mAP0.5= & \frac{1}{n} \sum _{c=1}^n \text {AP}_c \end{aligned}$$

4 $$\begin{aligned} mAP:0.95= & \frac{1}{T} \sum _{t=1}^T \frac{1}{n} \sum _{c=1}^n \text {AP}_c(t) \end{aligned}$$

where *n* is the number of classes in the respective task (e.g. pig detection or ear tag detection), *T* is the total number of IoU thresholds, *TP* is the number of true positive predictions, *FP* is the number of false positive predictions, *FN* is the number of false negative predictions and $\text {AP}_c$ is the average precision for class c. The $\text {AP}$ value summarizes the precision-recall curve into a single value by calculating the area under the curve. It is defined as:

5 $$\begin{aligned} AP_c = \sum _{k=0}^{m-1} \left[ \text {Recall}(k) - \text {Recall}(k+1) \right] \times \text {Precision}(k) \end{aligned}$$

where *m* is the number of confidence thresholds and *k* is the threshold index with $Recall(m) = 0$ and $Precision(m) = 1$^27^.

For the second assessment stage, we again computed the number of TP and FP, where a TP was assigned when an ID was correctly identified, and an FP was assigned when an ear tag was correctly detected, but the corresponding ID was incorrect. Based on this information, we calculated the Precision metric for both use cases and furthermore computed the number of FN and the Recall metric for the second use case only. This is because, in the first use case, the camera perspective makes it difficult even for a human evaluator to consistently pinpoint the exact moment when an ear tag ID should be recognized. Consequently, the number of FNs and, by extension, the recall metric could only be assessed in the second use case.

## Results and discussion

**Fig. 6:**
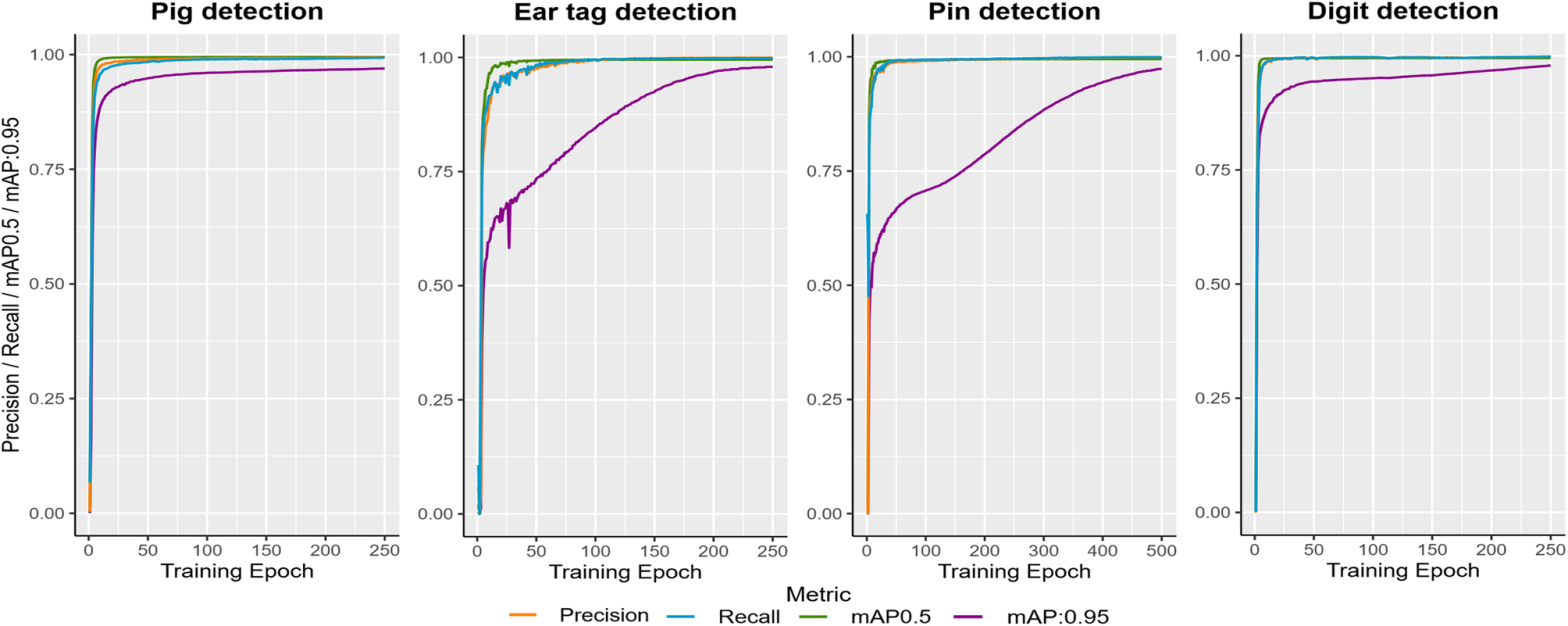
Model performances during training, assessed on the validation data after each training epoch.

For the first assessment stage, the training process of each model as well as the results of the model evaluation using the independent test datasets of each model are presented in Figure 6 and Table 2, respectively.

Monitoring the training process is crucial for detecting overfitting, a phenomenon where the model performs exceptionally well on the training data but shows decreased generalization performance on new data samples^28^. Measuring the model performance after each training cycle using the validation data reveals a steady increase in performance over time, excluding the presence of overfitting in the proposed work. In addition, the test data is used to evaluate the trained model’s performance on completely unseen data samples, ensuring an unbiased assessment, as the validation dataset is utilized for hyperparameter tuning and performance monitoring during the training process^29^. As can be seen in Table 2, all detection models achieve very high performance values in their respective tasks on the test data, consistently falling within the upper 0.9 range. This indicates a sufficient model state that can be further applied within the ID identification process.

**Table 2:** Results of the model evaluation using the independent test datasets.

Model evaluation	Precision	Recall	mAP0.5	mAP:0.95
Pig detection	0.994	0.993	0.995	0.970
Ear tag detection	0.999	0.998	0.995	0.979
Pin detection	0.998	0.999	0.995	0.974
Digit detection	0.998	0.997	0.995	0.979

Following the first evaluation stage, we proceeded to assess the combined model performance in the second evaluation stage, focusing on the two use case scenarios (see Section Model training and evaluation). Specifically for use case 1, we randomly selected and analyzed 500 ten-minute video sequences recorded at one frame per second (FPS), resulting in a total of 30,000 frames. Each frame was manually inspected to verify the correctness of the identified ear tag IDs. This evaluation focused on Precision, defined as the proportion of correctly detected and identified IDs (true positives) out of all detected IDs (true positives + false positives). Due to occlusion and pixelation, it was not feasible, even for human observers, to assess the Recall value in this scenario. Occlusion occurred naturally due to the animals’ behavior and posture, leading to partial or complete obstruction of objects, while pixelation resulted from the top-down camera perspective and the distance between the camera and the ear tags. These factors made it inevitable that not all ear tags were visible in every frame. In contrast, the second use case utilizes video data from an autonomous trail camera positioned at a close-up perspective. For this evaluation step, we again analyzed 500 randomly selected videos with a length of ten seconds and an FPS rate of 20 frames. The results of both use case scenarios are presented in Table 3.

**Table 3:** Identification results for the two use case scenarios.

Use Case	# IDs total	# IDs correct (TP)	# IDs incorrect (FP)	# IDs not detected (FN)	Precision	Recall
#1	45,515	45,410	104	-	0.996	-
#2	1,130	1,020	97	110	0.913	0.903

**Fig. 7:**
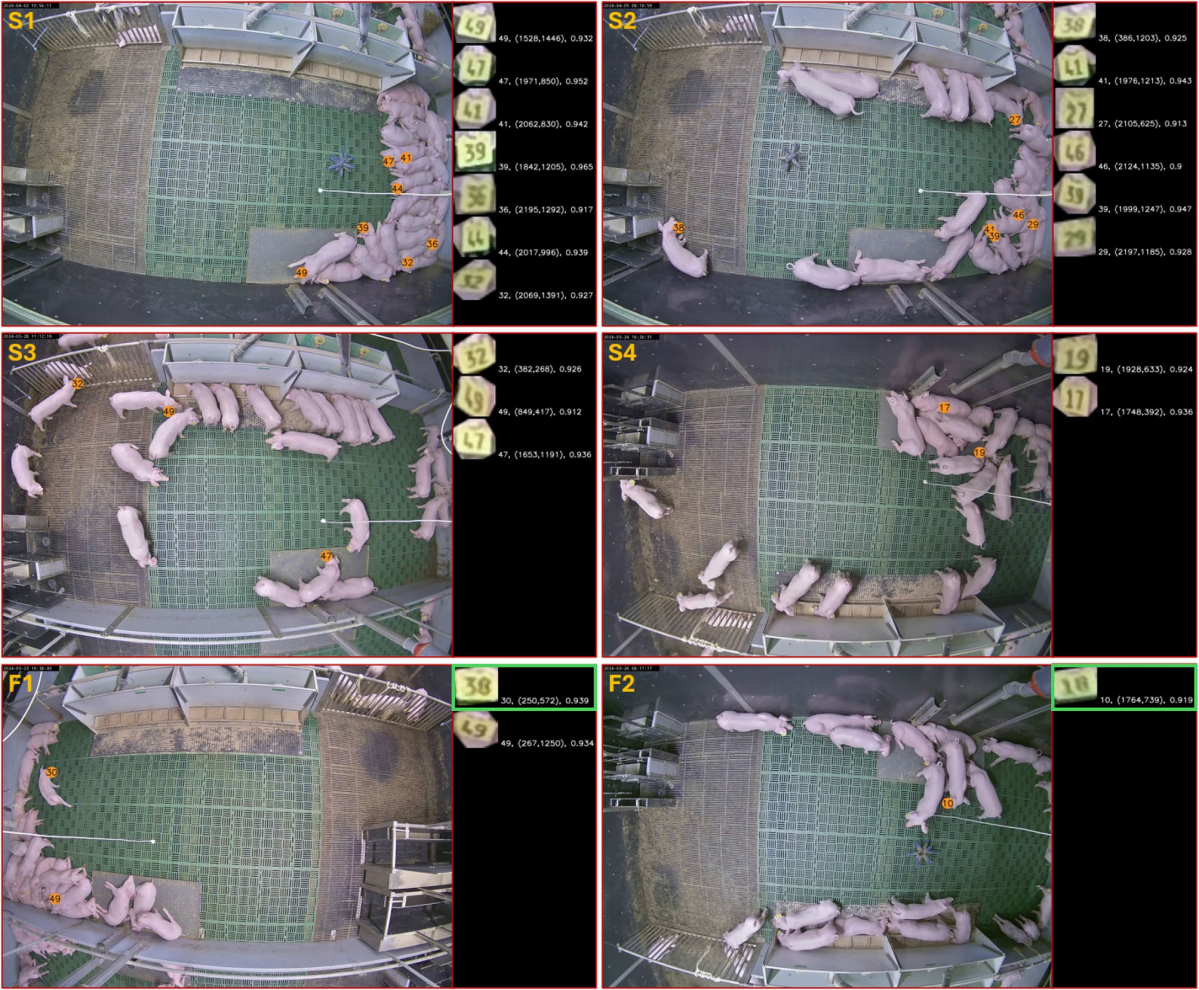
Examples of successful (S1–S4) and failed ID identification (F1 and F2) of images from Use Case 1. For illustration purposes, each subfigure displays the original RGB frame on the left, including detected IDs, and the rotation-corrected ID crops on the right, annotated with the ID prediction, center coordinates, and confidence score of each proposal. In failure cases, highlighted by the green box, motion blur was identified as the primary cause in most instances.

As shown in Table 3, when focusing on use case scenario 1, the proposed method achieves a high Precision value of 0.996, demonstrating that the majority of ID proposals were correctly identified. In the rare instances where an ear tag was detected but assigned an incorrect ID, we manually reviewed the model’s output and compared it with the ground truth image. In these cases, motion blur due to heavy animal movement and the distance of the camera to the ear tag was identified as the primary cause of unsuccessful identification (see Figure 7 F1 and F2). Nevertheless, in practical applications, it is essential that all animals are identified within a specific time window, as missed detections can result in a biased data representation and overlooked critical situations, ultimately leading to incorrect decisions at the management level. To tackle this issue–and considering that the Recall could not be calculated for Use Case 1–we analyzed ten one-hour recordings from a single pen taken over ten consecutive days (one recording per day; each video with an FPS rate of one frame). During this ten-hour observation period, we first calculated the frequency of occurrence of individual IDs and the average frame gap between consecutive detection events for each ID. To obtain the overall statistics, we summed the total frequency of each ID and computed the overall average interval as the mean of all individual intervals across IDs, which allowed us to further evaluate the detection performance of the proposed method for each ID. For the selected pen, a total of 24 animals were present, each assigned a consecutive ID ranging from 26 to 49. The corresponding results are presented in Table 4.

**Table 4:** Detection frequency and average frame gaps for a single pen from use case scenario 1. During the ten-hour observation period the majority of the animal IDs have been detected. IDs 44 and 48 (marked with *) were undetected in one video, which led to a considerable increase in the average frame gap.

ID	Frequency	Avg. Frame Gap	ID	Frequency	Avg. Frame Gap
26	5346	112	38	2919	18
27	1345	49	39	3245	13
28	4799	183	40	3995	17
29	2502	26	41	5323	17
30	7975	9	42	5985	10
31	4196	17	43	3645	25
32	909	61	44	3540	384*
33	3177	68	45	4052	12
34	4529	18	46	1707	36
35	4734	13	47	4784	13
36	4800	12	48	2581	388*
37	2409	26	49	3385	20

**Fig. 8:**
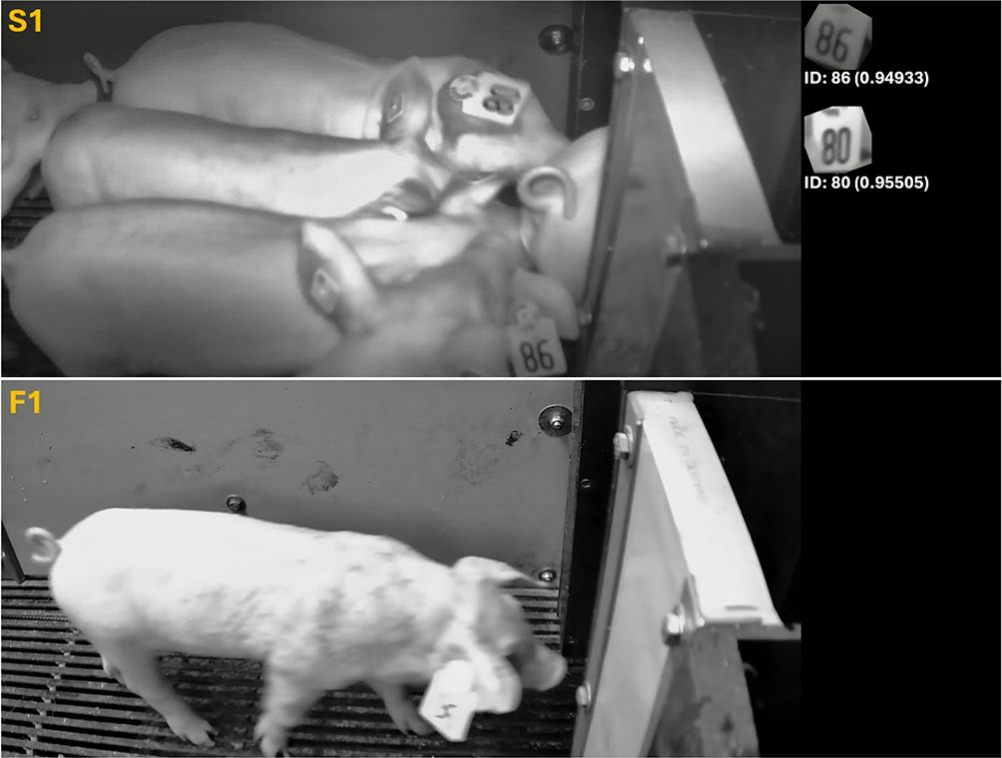
Example frames of a successful (S1) and a failed ID identification (F1) taken from Use Case 2. (S1) On the right side, two ear tag crops with ID proposals and confidence values in parentheses below the crop are shown. (F1) The ear tag with ID 96 was not detected due to a combination of overexposure of the image and motion blur of the animal.

In contrast, when focusing on the identification results of use case scenario 2 (Table 3), both the Recall and the Precision metric could be computed, as for this perspective it was possible to manually determine whether an ear tag ID should have been identified. Although this perspective was not learned during training the proposed method achieved Precision and Recall values of 0.913 and 0.903, respectively. Examples of successful and failure cases of both use cases are presented in Figure 7 and Figure 8, respectively.

When evaluating the method’s practical applicability, an important factor is its ability to cope with situations in which ear tags are placed in very close proximity – a circumstance frequently observed when animals rest closely together. Two examples of this behavior are shown in Figure 7 (S1 and S2) for the ID combinations 41 and 47 as well as 41 and 39, respectively. In such cases, multiple animals may lie together in a group, resulting in cropped body regions that contain more than one ear tag. The proposed approach is not constrained to a strict one-to-one mapping between pig detection and ear tag detection; instead, it accommodates the presence of multiple ear tags within a single cropped image. Each detected ear tag is individually processed for rotation correction and ID identification. In instances where duplicate IDs are identified over multiple pig instance detections, the system retains only the prediction with the highest confidence score. While this strategy does increase inference time, it enhances the method’s flexibility and applicability in real-world conditions.

**Fig. 9:**
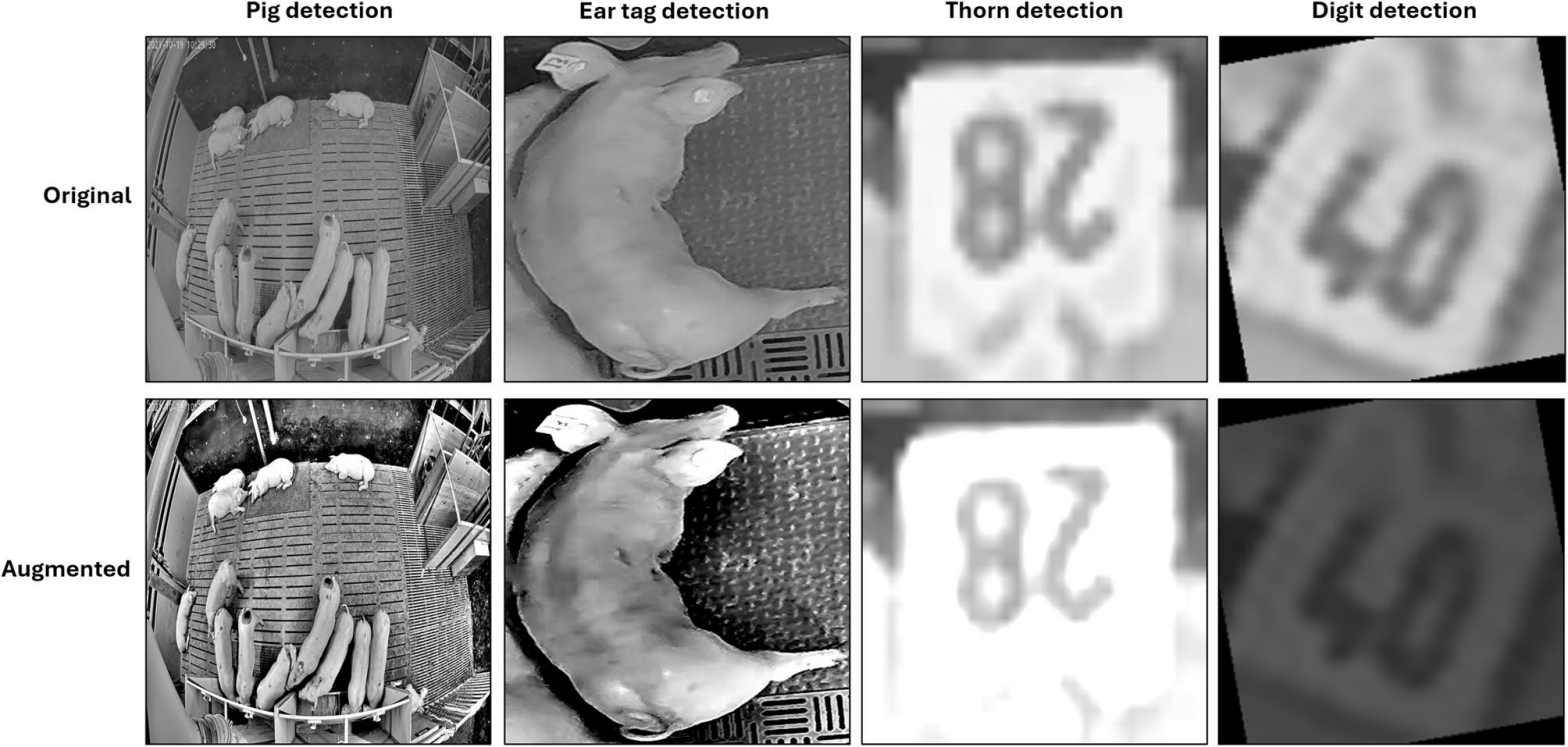
Example images from each test set showing the original image (top row) and the augmented images used to assess the model performance under challenging lighting conditions (bottom row).

Furthermore, since video data collected in practical husbandry environments is often affected by fluctuating lighting conditions^30^ – and because many CV methods are especially sensitive to such variations^31^ – we intentionally created overexposed and underexposed versions of the original test datasets. This served as an additional robustness check, allowing us to assess the performance of each analysis step in our approach under suboptimal lighting conditions. In this way, the individual model performance can be recorded and critical bottlenecks within the pipeline can be identified and directly addressed. To generate these variants, we employed a systematic image processing workflow: images were randomly modified by simulating either underexposure (by scaling pixel values with a factor between 0.4 and 0.6) or overexposure (by amplifying them with a factor between 1.1 and 1.3). As an additional variant, we changed the image structure and illumination characteristics of an image by applying a Single Scale Retinex (SSR)^32,33^ technique to enhance contrast and correct uneven illumination settings. In SSR, the logarithm of the original image is compared against the logarithm of its Gaussian-blurred counterpart, effectively normalizing lighting inconsistencies while preserving essential structural details. Example images for each dataset are provided in Figure 9 and the results of this additional robustness evaluation are shown in table 5.

**Table 5:** Results of the performance assessment for challenging lighting conditions.

Model evaluation	Precision	Recall	mAP0.5	mAP:0.95
Pig detection	0.997	0.996	0.995	0.976
Ear tag detection	0.986	0.963	0.989	0.891
Pin detection	0.996	0.991	0.995	0.888
Digit detection	0.998	0.996	0.995	0.970

As shown in Table 5, each model continues to achieve high performance values across all evaluation metrics even when illumination conditions are altered, with some results even slightly surpassing those obtained using the original, unaltered test data. One possible explanation for this enhanced performance is that the models were trained using augmented data, which involved random modifications to hue, saturation, brightness, and contrast, while evaluation was conducted on unaltered images. As a result, the models may have developed greater robustness to a range of lighting variations during training, enabling effective generalization to conditions such as overexposure, underexposure, and broader changes in image composition. Consequently, as part of their generalization ability, the object detection models may be better equipped to focus on invariant, task-relevant features. In addition, this improvement could be due to the fact that certain illumination adjustments - such as over- or underexposure or the application of SSR - could help improve visual discriminators or reduce noise in low-light situations or scenes with already inconsistent lighting conditions, which are also part of the original test dataset.

In contrast to alternative animal identification methods that rely on color filtering of the ear tag itself, which is often unsuitable under low-light or nighttime conditions, or approaches based on more specialized ear tag codings, which increase complexity and require an additional matching step of the ear tag and ID information, the proposed method leverages only grayscale information and a refined CNN-based feature extraction pipeline, allowing the continuous monitoring of specific pen areas and the direct determination of animal specific IDs. Although other identification strategies, such as facial recognition^34^ or muzzle-print analysis^35^, have shown promising potential for certain species like cattle, they can be impractical for species with less-prominent features or in settings that require strict camera angles. Additionally, incorporating more sophisticated sensors or higher-resolution cameras to tackle these drawbacks further increases complexity and cost^36^. In this regard, our method remains robust across various lighting conditions, does not require specialized tag geometry, and thus represents a practical, cost-effective solution for commercial pig farms seeking reliable individual identification.

One of the main challenges encountered in the proposed ear tag detection method is motion blur, which increases the number of unreadable ear tags, particularly when using the top-down camera perspective. In such cases, most false positive results stem from the misinterpretation of the digits zero and eight, as their similar shapes make them difficult to distinguish under blurred conditions. However, despite this limitation, the overall detection performance remains strong. Since the current system employs two-digit IDs but could theoretically be extended to more digits, future work could mitigate this issue by either excluding one of the two problematic digits or modifying their visual design to enhance their distinctiveness and reduce confusion.

Since one of the goals of this study is to provide the underlying datasets, further research should be encouraged to expand the scale of ear tag-based animal identification. A robust animal detection and identification system has the potential to facilitate more advanced applications, such as longterm animal tracking in precision livestock farming. Here, maintaining consistent animal identities in video-based monitoring remains one of the most critical challenges in precision livestock farming, as individuals can undergo substantial appearance changes, suffer frequent occlusions, and exhibit complex group dynamics. This limitation directly impacts the reliability and scalability of automated systems aimed at continuously tracking individual animals’ health and behavior. Therefore, the development of consistent re-identification methods is a major task in the computer vision area^37,38^. As illustrated in the first use case scenario, the robust identification of ear tag IDs can serve as an additional step within a multi-object tracking system to validate the accuracy of existing tracks, detect false tracks, and determine ID-switches.

Future work could explore the modularity of the proposed pipeline, which consists of four distinct object detection models designed to detect different aspects of the image. This modular structure allows for the replacement of specific components, such as the pig detection model, to extend the approach to other livestock species, including cattle, sheep, and goats. By adapting the detection models, this framework can be generalized to a broader range of animal identification applications. Furthermore, upcoming research will focus on integrating this method within tracking applications to address the challenge of re-identification. By incorporating temporal data and movement patterns, the system can enhance its ability to track individual animals over time, ensuring consistent identification even in dynamic farm environments. This integration will be crucial for improving livestock monitoring systems, supporting real-time animal tracking, and enhancing overall farm management efficiency.

## Conclusion

This study leverages the efficient feature extraction capabilities of convolutional neural networks to analyze animal and ear tag information, ultimately generating an ID proposal for identifying individual pigs. By utilizing grayscale image data and incorporating an additional detection step for the ear tag pin, the proposed method remains effective for both daytime and nighttime applications, enabling continuous monitoring from various camera angles and distances. Additionally, a key contribution of this work lies in the publication of the underlying datasets, particularly for ear tag, pin, and digit detection. By making these datasets publicly available, this study supports the research community by providing valuable resources for training and benchmarking object detection models, fostering further advancements in animal identification and precision livestock farming.

## Data Availability

The datasets generated in the course of this study are available at the opendata@uni-kiel research data repository of the Christian-Albrechts-University Kiel. They are accessible through the following link: https://opendata.uni-kiel.de/receive/fdr_mods_00000115?accesskey=p8mePt8lB8RnveYnIjCtR8SO5o8eqot8.
